# Characterization of Five Novel Mitoviruses in the White Pine Blister Rust Fungus *Cronartium ribicola*

**DOI:** 10.1371/journal.pone.0154267

**Published:** 2016-05-19

**Authors:** Jun-Jun Liu, Danelle Chan, Yu Xiang, Holly Williams, Xiao-Rui Li, Richard A. Sniezko, Rona N. Sturrock

**Affiliations:** 1 Pacific Forestry Centre, Canadian Forest Service, Natural Resources Canada, Victoria, BC, V8Z 1M5, Canada; 2 Pacific Agri-Food Research Centre, Agriculture and Agri-Food Canada, Summerland, BC, V0H 1Z0, Canada; 3 USDA Forest Service, Dorena Genetic Resource Center, Cottage Grove, Oregon, 97424, United States of America; Ruhr-University Bochum, GERMANY

## Abstract

The white pine blister rust (WPBR) fungus *Cronartium ribicola* (J.C. Fisch.) is an exotic invasive forest pathogen causing severe stem canker disease of native white pine trees (subgenus *Strobus*) in North America. The present study reports discovery of five novel mitoviruses in *C*. *ribicola* by deep RNA sequencing. The complete genome of each mitovirus was determined by rapid amplification of cDNA ends (RACE) and reverse transcriptase-polymerase chain reaction (RT-PCR). A single open reading frame (ORF) encoding a putative RNA-dependent RNA polymerase (RdRp) was detected in each of the viral genomes using mitochondrial genetic codes. Phylogenetic analysis indicated that the *C*. *ribicola* mitoviruses (CrMV1 to CrMV5) are new putative species of the genus *Mitovirus*. qRT-PCR and RNA-Seq analyses revealed that viral RNAs were significantly increased in fungal mycelia in cankered pine stems compared to expression during two different stages of spore development, suggesting that viral genome replication and transcription benefit from active growth of the host fungus. CrMVs were widespread with relatively high levels of minor allele frequency (MAF) in western North America. As the first report of mitoviruses in the Class *Pucciniomycetes*, this work allows further investigation of the dynamics of a viral community in the WPBR pathosystem, including potential impacts that may affect pathogenicity and virulence of the host fungus.

## Introduction

The genus *Mitovirus* is a group of fungal viruses (mycoviruses) belonging to the family *Narnaviridae* [1]. Mitoviruses do not form true viral particles and only have a linear 2.3–3.1 Kb single-stranded positive RNA genome (ssRNA(+)) that encodes a single protein—the RNA dependent RNA polymerase (RdRp) [2]. Mitoviruses are proposed to replicate their genomes in the form of double-stranded RNA (dsRNA). They are detected predominantly in the mitochondria of host cells except two mitoviruses infecting *Thanatephorus cucumeris* and *Rhizophagus clarus*, which have the potential to replicate in the cytosol and in mitochondria [3, 4].

Since 1994, when the first genome sequencing of a mitovirus in the chestnut blight ascomycete fungus *Cryphonectria parasitica* was completed [5], an increasing number of plant pathogenic fungi have been reported as hosts of mitoviruses [3–23]. While most of the fungi affected by mitoviruses belong to the phylum Ascomycota, a few fungi in Basidiomycota and one arbuscular mycorrhizal (AM) fungus also host these viruses (S1 Table).

Growing evidence suggests that mitoviruses can decrease the pathogenicity and growth of their host fungi by interfering with the normal function of the host mitochondria [16, 24–26]. Indeed, mycovirus-mediated hypovirulence, which is characterized by slow spore development and reduced growth rates (i.e. attenuated pathogenicity), has been explored as a strategy for biological control of plant pathogenic fungi [27, 28].

*Cronartium ribicola* (J.C. Fisch) is a fungal pathogen that causes white pine blister rust (WPBR) in five-needle pine species of all ages in forests around the world. WPBR is considered to be one of the most important forest diseases in North America since it was introduced from Europe at the turn of the 20th century. Management of this exotic forest disease is challenging despite efforts focused on breeding for resistance in native, North American-white pine species. As an obligate biotrophic fungus, *C*. *ribicola* requires two host plant species (five-needle *Pinus* spp. and *Ribes* spp.) to complete its life cycle. The WPBR life cycle includes development of five different types of spores: pycniospore and aeciospore stages on five-needle pines, and urediniospore, teliospore, and basidiospore stages on *Ribes*. We recently reported on candidate pathogenicity and virulence genes for *C*. *ribicola*, which we determined by profiling fungal transcriptomes expressed during tree-fungus interactions [29]. While different avirulent and virulent isolates have been reported in *C*. *ribicola* populations [30], biological characteristics of the fungus such as pathogenicity and interaction with other microorganisms are poorly understood. This is largely due to the obligate biotrophic nature of the fungus.

The objective of the present study was to use deep mRNA sequencing/RNA-Seq for discovery and characterization of any mycoviruses present in the WPBR pathosystem. Here we report for the first time a group of mycoviruses infecting *C*. *ribicola*. The viruses are novel distinct species of the genus *Mitovirus*, tentatively named as *Cronartium ribicola* mitoviruses 1, 2, 3, 4, and 5 (CrMV1 to CrMV5).

## Materials and Methods

### Isolates of *C*. *ribicola*

*C*. *ribicola* aeciospores were collected from intact aecial blisters occurring on infected stems of western white pine (*Pinus monticola*, Douglas ex D. Don) at Coombs, Vancouver Island, British Columbia (BC), Canada in May 2013. Aeciospores were used to inoculate black currant plants (*Ribes nigrum*, cultivar Ben Nevis) in a greenhouse. When urediniospores began to develop on current plants, urediniospores (containing some mycelia) were collected from the undersides of infected *Ribes* leaves. Additional *C*. *ribicola* isolates were collected in Oregon (OR), USA from infected *P*. *monticola* stems at the mycelium growth stage or the aeciospore stage, as described previously [29]. No specific permission was required for us to sample aeciospores in these locations in Canada and USA. Canadian Food Inspection Agency (CFIA) issued a permit for Pacific Forestry Centre to import *C*. *ribicola* samples from OR, USA. A total of 15 *C*. *ribicola* isolates were collected, including nine avirulent (*avcr2*) and six virulent (*vcr2*) isolates originating from susceptible (*cr2/cr2*) or major gene resistant (*Cr2/-*) western white pine stems, respectively (Table 1). *C*. *ribicola vcr2* isolates are able to overcome the western white pine *Cr2* gene for successful infection of seedlings with *Cr2/-* genotypes. All samples were harvested in liquid nitrogen and immediately stored at -80°C before RNA extraction.

**Table 1:** *Cronartium ribicola* isolates and mitoviral contigs detected in their transcriptomes de-novo assembled in RNA-seq analysis.

Fungal isolates	Contig no. (n)	Contig length (bp)	Top hit species in BLASTx (*)	Lowest E-value	Total contig (n)	Total RNA-Seq reads (**)	Pathotype and region	Fungal life-cycle stage	RNA-Seq	qRT-PCR
**BC-a6**	0	na	na	na	15,510	48.1	avcr2, BC	Aeciospore	Yes	Yes
**BC-a20**	2	558; 208	OMV5; SsMV6	1E-07 ~ 6E-08	15,830	33.5	avcr2, BC	Aeciospore	Yes	Yes
**BC-a28**	4	1,348; 535; 308; 204	GaMVS2; OMV5; SsMV6	5E-06 ~ 8E-25	20,118	33.1	avcr2, BC	Aeciospore	Yes	Yes
**BC-u48**	6	704; 594; 462; 330; 302; 262	GaMVS2; OMV5; OMV6	7E-11 ~ 2E-35	29,908	38.0	avcr2, BC	Urediniospore	Yes	Yes
**BC-u3**	2	1,155; 369	GaMVS2; TbMV	6E-06 ~ 7E-34	12,986	20.9	avcr2, BC	Urediniospore	Yes	Yes
**BC-u2a**	7	1,988; 1,492; 1,469; 1,198; 1,126; 697; 439	GaMVS2; OMV4; OMV5; OMV6	6E-13 ~ 5E-41	37,180	32.9	avcr2, BC	Urediniospore	Yes	Yes
**OR-sus1**	9	2,531; 2,511; 1,265; 1,217; 999; 756; 404; 260; 230	GaMVS2; OMV4; OMV5; OMV6	2E-08 ~ 5E-47	109,185	75.9	avcr2, OR	Cankered stem	Yes	Yes
**OR-sus2**	6	2,028; 1,711; 1,568; 1,401; 436; 344	GaMVS2; OMV4; OMV5; OM-6; SsMV6	5E-06 ~ 3E-42	96,684	89.8	avcr2, OR	Cankered stem	Yes	Yes
**OR-sus3**	5	2,462; 2,383; 2,375; 1,844; 1,124	GaMVS2; OMV4; OMV5; OMV6; TbMV	1E-37 ~ 4E-47	83,452	59.7	avcr2, OR	Cankered stem	Yes	Yes
**OR-res1**	na	na	na	na	na	na	vcr2, OR	Cankered stem	no	Yes
**OR-res2**	na	na	na	na	na	na	vcr2, OR	Cankered stem	no	Yes
**OR-res3**	na	na	na	na	na	na	vcr2, OR	Cankered stem	no	Yes
**OR-a1**	na	na	na	na	na	na	vcr2, OR	Aeciospore	no	Yes
**OR-a2**	na	na	na	na	na	na	vcr2, OR	Aeciospore	no	Yes
**OR-a3**	na	na	na	na	na	na	vcr2, OR	Aeciospore	no	Yes
**Healthy pine stem**	0	na	na	na	95,727	123.1	na	na	Yes	Yes

Note

(*) Transcriptome of each fungal isolate was used as a query against the NCBI viral protein database at E value cut-off (e-5). The top hit species were *Gremmeniella abietina* mitovirus (GaMV), *Ophiostoma* mitovirus (OMV), *Sclerotinia sclerotiorum* mitovirus (SsMV), and *Thielaviopsis basicola* mitovirus (TbMV).

(**) RNA-seq reads were counted in million and deposited in GenBank under SRA numbers SRR1574690-SRR15774692, SRR1583540, SRR1583545, SRR1583552, SRR15835557-SRR1583559, and SRR3273235-SRR3273237.

### *In silico* discovery of mycoviral sequences

RNA-Seq raw reads from nine *avcr2* isolates and a negative control (healthy *P*. *monticola* stems without *C*. *ribicola* infection) from previous studies [29, 31] were used for *in silico* discovery of mycoviral sequences (Table 1). These RNA-Seq raw reads are accessible in GenBank under SRA numbers SRR1574690-SRR15774692, SRR1583540, SRR1583545, SRR1583552, SRR15835557-SRR1583559, and SRR3273235-SRR3273237. After trimming of adaptor and low-quality sequences, deep mRNA sequencing reads were *de novo* assembled using CLC Genomics Workbench version 5.5 with graph parameters of automatic word size and automatic bubble size (CLC bio, QIAgen, Aarhus, Denmark). Putative viral sequences were identified from the *C*. *ribicola* transcriptomes by BLASTx analysis against a data set of the Viral RefSeq (viral.1.protein.faa.gz) downloaded from the National Center for Biotechnology Information (NCBI, Bethesda, Maryland, USA). Possible overlapping contigs from different fungal isolates were re-assembled into longer consensus sequences using the CAP3 program with default settings [32].

### Viral transcript analysis

To determine the relative prevalence of viral ssRNA(+) in *C*. *ribicola* samples, trimmed reads from fungal isolates were mapped to the viral genomes and only paired reads (fragments) were counted in mapping with a mismatch cost of 1, indel cost of 3, length fraction of 0.95, and similarity fraction of 0.95 using CLC Genomics Workbench v5.5. Fragments per kilobase of exon per million reads mapped (FPKM) was used to evaluate the relative level of ssRNA(+) or transcripts. Total RNA-Seq reads in each fungal isolate were calculated as those that were mapped to the *C*. *ribicola* reference transcriptome [29]. One-tailed independent t-tests (*P* <0.05) were used to assess differences in viral transcript levels among fungal isolates or among different viruses inside the same fungal isolate.

### Detection of single nucleotide polymorphisms (SNPs) in the viral genomes

*In silico* SNP detection was performed by mapping RNA-Seq paired-end reads back to the targeted sequences using CLC Genomics Workbench v5.5 with the same parameters used in FPKM calculation. SNPs were detected using quality-based variation detection at the following parameters: neighborhood radius = 5, maximum gap and mismatch count = 1, minimum neighborhood quality = 20, minimum central quality = 20, ignore non-specific matches = yes, ignore broken pairs = no, minimum coverage = 10, minimum variant frequency (%) = 5.0, maximum expected alleles = 2, require presence in both forward and reverse reads = yes, genetic code = 3 yeast mitochondrial.

### Sanger sequencing analysis of viral RNA genomes

To verify *de novo* assembled mitoviral sequences, oligo-nucleotide primers (S2 Table) were designed based on *in silico* sequences. Total RNA was used for cDNA synthesis with viral genome-specific primes using a SuperScript® III First-Strand Synthesis System (Life Technologies; Burlington, ON). The *C*. *ribicola avcr2* isolate collected in BC, Canada (BC-u3) was used for PCR amplification of viral cDNA fragments. The 5’- and 3’-ends of the viral genomes were determined using a method of rapid amplification of complementary DNA ends (RACE). Because there is a dsRNA stage in the replication cycle of mitoviruses [5], we used an Invitrogen 5′ RACE System (Invitrogen, Grand Island, NY) following the manufacturer’s protocol to determine the terminal sequences of both the positive and the negative strand of the dsRNA.

The RT-PCR products were separated on a 1% (w/v) agarose gel in TAE buffer. DNA fragments with expected sizes were purified using a MinElute gel extraction kit (Qiagen) and cloned into the pGEM-T Easy vector (Promega). Recombinant clones were selected after transformation of ligation mix into *E*. *coli* DH5α competent cells. The insert sequences of recombinant plasmids were determined by Sanger sequencing using T7and SP6 universal primers, or internal primers as needed on an ABI 3730XL sequencer. DNA sequences were assembled into overlapping contigs for each individual genome using the software Sequencer (Gene Codes, Ann Arbor, MI). Complete nucleotide sequences of the *C*. *ribicola* mitoviruses were deposited in GenBank under accession numbers KT921179 to KT921183.

Open reading frames (ORFs) encoding putative proteins were found using the NCBI ORF Finder. Searches for protein domains were performed using NCBI conserved domain database (CDD). Potential secondary structures of 3′-terminal and 5′-terminal nucleotide sequences of viral genomes (positive strand) were predicted and the free energy (ΔG) was estimated using MFOLD software [33]. Multiple sequence alignment was conducted for the deduced amino acid sequences of the RdRp proteins using CLUSTAL-Omega at EMBL-EBI website. On the basis of aligned sequences, phylogenetic trees were further constructed using the neighbour-joining method with the MEGA version 6.0 program [34].

### Quantitative reverse-transcriptase PCR (qRT-PCR) analysis

qRT-PCR was used to evaluate virus prevalence in all of 15 *C*. *ribicola* isolates collected in the present study (Table 1). The first strand cDNA was synthesized from 2 μg of total RNA (including viral ssRNA and dsRNA genomes) as template with random primers using a Superscript® VILO™ master mix kit (Life Technologies). Mitovirus species-specific primers were designed using the software PrimerExp (ABI) (S2 Table), and three replicates per sample were run on an Applied Biosystems 7500 Fast Real-time PCR System (Life Technologies). Genomic DNA, no reverse transcriptase and water samples were run as negative controls for each primer pair. *C*. *ribicola α-tubulin* was included as an internal control for normalization of fungal RNA level across isolates. Relative RNA level was analyzed using Expression Suite Software v1.0.3 (Life Technologies) with the 2^−ΔΔCT^ method as described previously [29]. One-tailed independent t-tests (*P* <0.05) were used to assess difference of RNA levels between fungal isolates. Pearson correlation analysis was performed to compare fold changes of RNA levels measured by qRT-PCR and FPKM analyses. To confirm prevalence of the viruses as detected by qRT-PCR, regular RT-PCR was performed to amplify cDNA fragments in the size range indicated by Sanger sequencing and RNA-Seq data for each viral genome across 15 fungal isolates.

## Results

### Detection of Mitovirus-like sequences by deep mRNA sequencing

Deep mRNA sequencing yielded about 21.9 to 89.9 million 100-bp PE reads from each of the nine *C*. *ribicola avcr2* isolates (Table 1). The number of cDNA contigs *de novo* assembled from RNA-Seq reads by the CLC assembly algorithm ranged from 12,986 to 109,185. The contig data set was further used as a BLASTx query to search against the NCBI dataset of viral protein sequences. In total, 41 putative mitovirus-like sequences were identified in eight of the nine *C*. *ribicola avcr2* isolates. Only isolate BC-a6, an aeciospore sample, lacked any identified mitoviral sequences (Table 1). The 41 mitovirus-like sequences had best homology hits with *G*. *abietina* mitovirus S2 (GaMVs2), *Ophiostoma* mitoviruses (OMV4, OMV5, and OMV6), *Sclerotinia sclerotiorum* mitovirus (SsMV), and *Thielaviopsis basicola* mitovirus (TbMV), with E values ranging from 5E-06 to 4E-47. In contrast, no mitovirus-like contigs were found in the transcriptome of an uninfected *P*. *monticola* stem in the BLASTx analysis (Table 1). These results suggest that the mitovirus-like transcripts might originate from *C*. *ribicola*. The overlapping regions among the contigs from different *C*. *ribicola* isolates showed high identities (98%~100%). Re-assembly of the overlapping contigs from different fungal isolates using the CAP3 program generated five consensus mitovirus-like sequences.

### Molecular characterization of CrMV based on Sanger sequencing

To clone full-length sequences of partial mitovirus-like cDNA fragments detected by RNA-Seq analysis, we designed six primers for each consensus sequence (S2 Table): one pair of primers for internal fragment cloning, and four primers for 3’-RACE and 5’-RACE. Sequences of PCR fragments were determined by Sanger sequencing. Assembly of cDNA sequences from the BC-u3 isolate formed five complete sequences, ranging from 2,471 nt to 2,715 nt (Table 2). In contrast, no sequences were amplified from *C*. *ribicola* genomic DNA, indicating that the five viral sequences did not originate from the *C*. *ribicola* genome. We succeeded in re-sequencing all 3’-terminal regions by a 5’-RACE analysis, indicating that the dsRNA was a replication form of the targeted mitovirus-like sequences in the fungus.

**Table 2:** Sequence characterization of *Cronartium ribicola* mitoviruses (CriMV) based on Sanger sequencing of cDNA clones.

Virus ID	Total length	5'UTR	3'-UTR	RdRp (aa)	5'-Small ORF (aa) ^(^^*^	A/U content
**CrMV1**	2,715	1–234	2,638–2,715	235–2, 637 (800)	40–234 (64)	57.30%
**CrMV2**	2,471	1–277	2,348–2,471	278–2,347 (689)	nil	58.90%
**CrMV3**	2,522	1–237	2,380–2,522	238–2,379 (713)	77–217 (46)	62.10%
**CrMV4**	2,479	1–184	2,351–2,479	185–2,350 (721)	nil	59.50%
**CrMV5**	2,631	1–207	2,526–2,631	208–2,526 (772)	3–248 (81)	57.40%

* Open reading frame (ORF) was detected using the NCBI ORF Finder.

A large open reading frame (ORF) was detected using yeast mitochondrial genetic codes in each viral full-length nucleotide sequence. When the universal standard genetic codes were used, much shorter ORFs corresponding to fractured segments of RdRp were found that contained several intra-ORF UGA codons. This result suggested that viral translation was localized in the *C*. *ribicola* mitochondria. A preliminary BLASTp search against the conserved Domains Database (CDD) revealed only one conserved domain, the viral RdRp domain (pfam05919: Mitovir_RNA_pol), in the putative proteins encoded by the large ORFs in the five full-length viral sequences. Therefore, we proposed these viral sequences to be *C*. *ribicola* mitoviruses (CrMV1 to CrMV5).

The CrMV genomes showed a rich A + U content ranging from 57.3% (CrMV1) to 62.1% (CrMV3). The length of the putative RdRp varied from 689 (CrMV2) to 800 (CrMV1) amino acid residues (Table 2). CrMV1 and CrMV4 genomes began with AUA as the start codon, while other three CrMV genomes had a typical AUG initiation codon for their RdRp-coding regions. In addition, a small ORF was detected upstream of the RdRp coding region in CrMV1 (64 aa), CrMV3 (46 aa), and CrMV5 (81 aa). The putative proteins encoded by these small ORFs showed no homology hit in the NCBI nr database, a result similar to that reported previously in a *B*. *cinerea* mitovirus [35].

### Phylogeny of CrMVs

**Fig 1:**
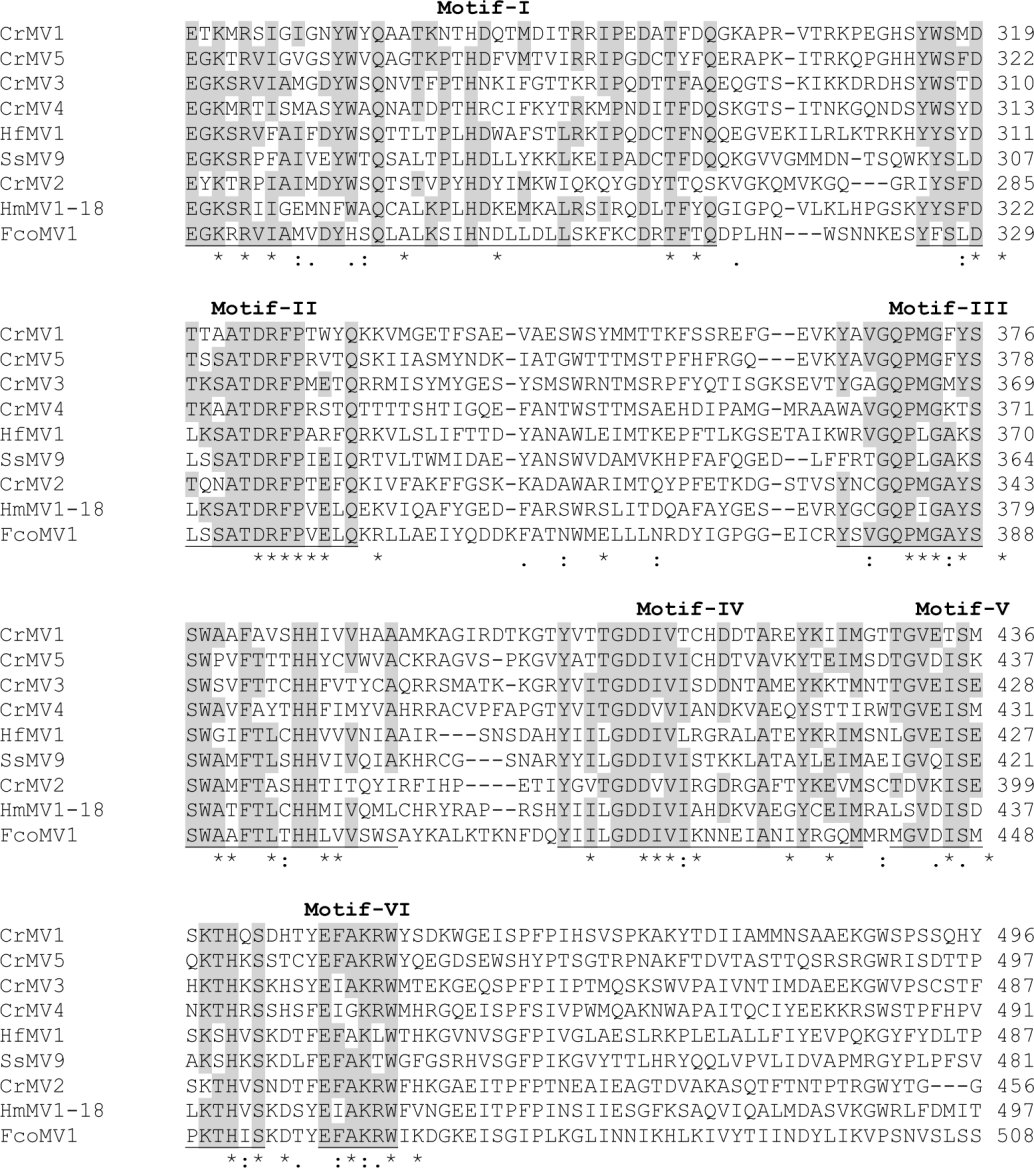
Sequence alignment analysis of the putative RNA-dependent RNA polymerases (RdRp) of *Cronartium ribicola* mitoviruses (CrMV1 to CrMV5). Six conserved RdRp motifs (I to VI) among Cri-MVs are labelled according to previous report (Hong et al. 1999). Alignment analysis was performed using CLUSTAL program, and it included four other mitovirus species: FcoMV1 (BAQ36630), HfMV1 (AIU44705), HmMV1-18 (BAD72871), and SsMV1 (YP_009121785). Symbols (*), (:), and (.) below the sequences are used to indicate identical amino acids, or residues with chemical-similarities at higher and lower levels respectively.

Alignment analysis revealed that CrMV genomes shared nucleotide sequence identities from 45.66% (CrMV2 vs. CrMV4) to 54.82% (CrMV3 vs. CrMV5), while RdRp protein sequence identities ranged from 23.66% (CrMV4 vs. CrMV2) to 35.13% (CrMV1 vs. CrMV5). When RdRp amino acid sequences of CrMVs were compared with other mitoviruses, sequence identities ranged from 15.05% (CrMV5 vs. *Ophiostoma* mitovirus 1c under GenBank accession no. AGT55876.1) to 28.37% (CrMV2 vs. *H*. *mompa* mitovirus 1–18 A under GenBank accession no. BAD72871.1). CrMV RdRp proteins shared six conserved motifs (I to VI) with other mitoviruses (Fig 1). These motifs are widely detected in the genus *Mitovirus* [26], with motif-I as a structural feature of the genus *Mitovirus* [12] and motif-V as the GDD region universal among RdRps from different RNA virus families [36].

**Fig 2:**
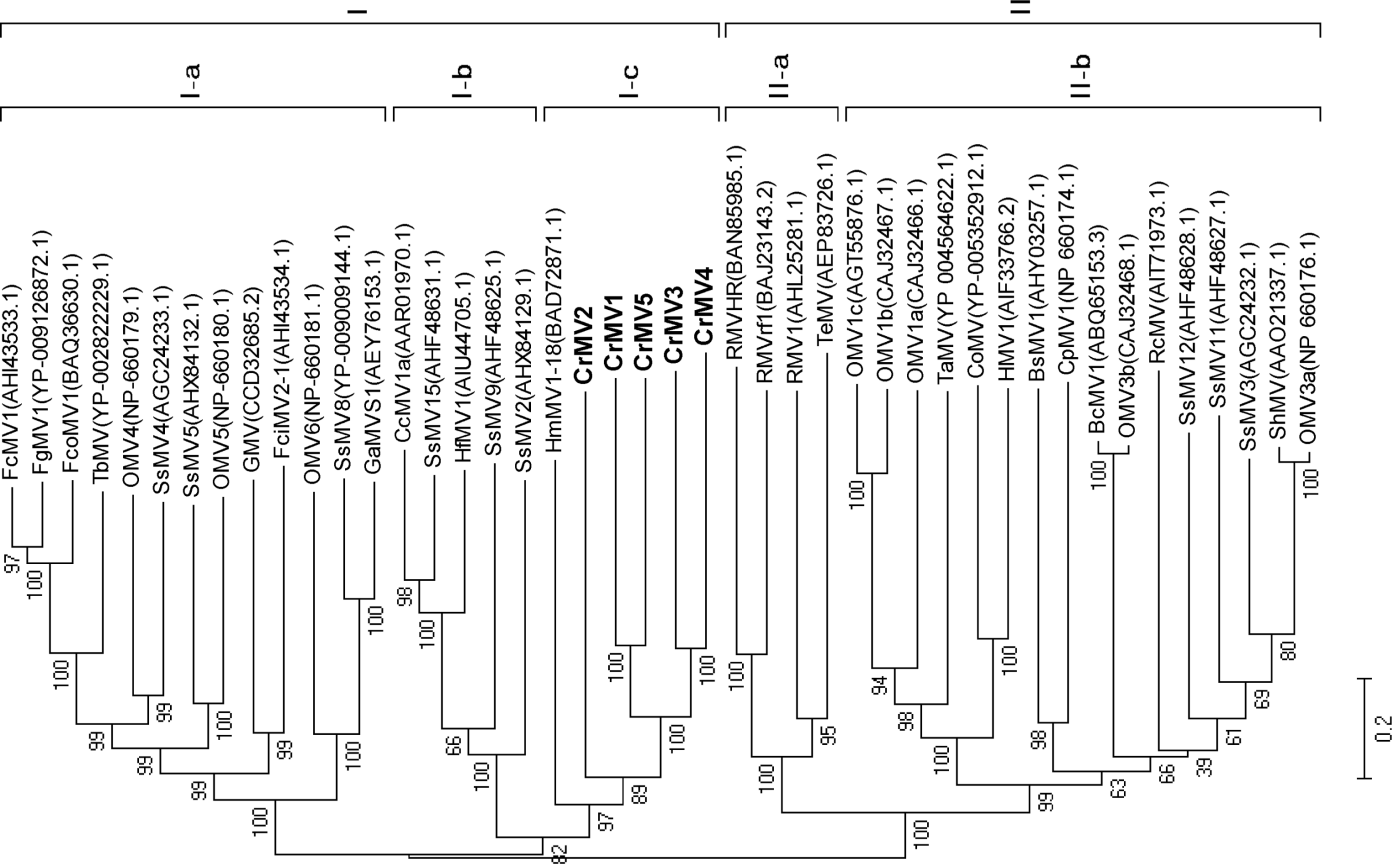
Phylogenetic tree analysis of the novel *Cronartium ribicola* mitoviruses (CrMV1 to CrMV5) and representative species (with GenBank accession numbers) of the genus *Mitovirus*. Neighbor-joining method was used for the phylogenetic analysis based on full-length alignment of RNA-dependent RNA polymerases (RdRp). Bootstrap support resulting from 1000 replicates is shown on the internodes and branch lengths correspond to genetic distance; the scale bar at lower left corresponds to a genetic distance of 0.2 for the RdRp sequences.

Because RdRp genes are highly conserved among RNA viruses [36], a phylogenetic tree was constructed for CrMVs. On the tree we also included representatives of full-length sequences of members of the genus *Mitovirus* that were downloaded from GenBank. Phylogenetic analysis based on alignment of RdRp proteins showed that mitoviruses were grouped into two main clusters (cluster-I and cluster-II), a pattern previously reported [2]. All five CrMVs formed a diverse, but monophyletic subcluster (I-c) along with HmMV1-18 in the cluster-I (Fig 2). Deep distances among CrMVs suggest that these sequences represent five new species of the genus *Mitovirus*.

### Predicted secondary structures for CrMV terminal sequences

**Fig 3:**
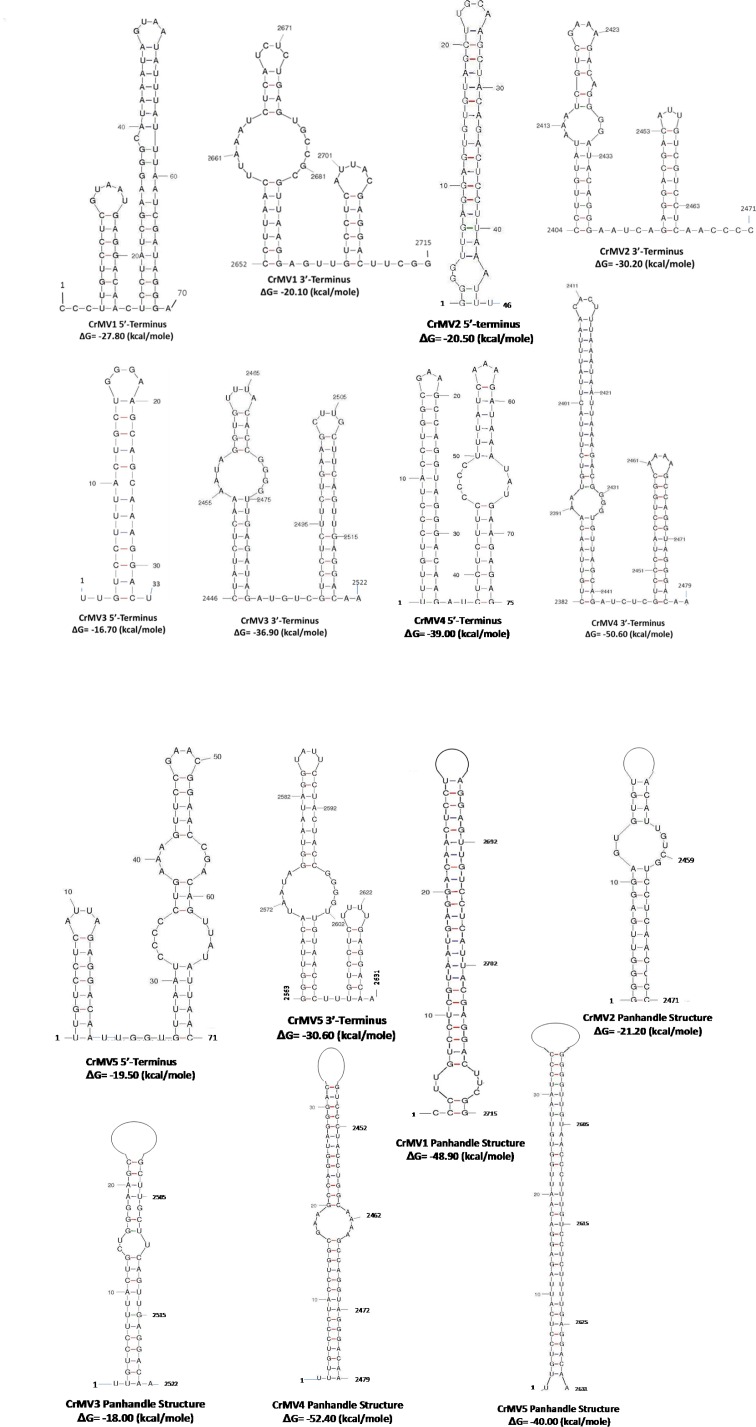
Secondary structures in the 5′- and 3′-terminal regions of *Cronartium ribicola* mitoviruses (CrMV1 to CrMV5) as predicted using the MFOLD program. Each number indicates the nucleotide position from the 5′ end of the viral ssRNA(+) genome. Stem-loop structures were predicted by *in silico* hybridizations of the 5′- or the 3′-terminal sequences. Panhandle structures were predicted between conservative and complementary 5’- and 3’- terminal sequences.

Mitoviruses can be potentially folded into stable stem-loop structures at both the 5′- and 3′-terminal sequences. Moreover, the 5′- and 3′-terminal sequences of some mitoviruses were inverted complementary and predicted to form a panhandle structure [15]. Because of the importance of these secondary structures in stabilizing RNA viral genomes and initiating genome replication and transcription [15], we examined genome sequences of CrMVs for potential secondary structure using the MFOLD program. All five CrMV genomes were predicted with potentials to form stem-loop structures at both 3’- and 5’-terminal sequences. The free energy (ΔG) values were calculated in a range of -16.7 (5’-terminus of CrMV3) to -50.6 kcal/mole (3’-terminus of CrMV4). Panhandle structures were predicted as formed by inverted terminal complimentary sequences at 5′- and 3′-end for each CrMV genome (Fig 3). The ΔG values of these panhandle structures were calculated at -18.0 (CrMV3) to -52.4 kcal/mole (CrMV4). The ΔG values suggest that the stem-loop and panhandle secondary structures may be stable for each CrMV genome.

### CrMV distribution in *C*. *ribicola* isolates

qRT-PCR was performed to evaluate presence and abundance of the viral RNA sequences, including both ssRNA and dsRNA, in different *C*. *ribicola* isolates. CrMV specific primers were designed at the C-terminal variable regions of RdRp for each mitovirus. One pair of primers for the *C*. *ribicola α-tubulin* gene was included as an internal control for RNA extraction, cDNA synthesis, and normalization of host cells in qRT-PCR analysis (S2 Table). CrMV1 to CrMV5 were detected in all 15 fungal isolates of the selected three stages of the fungal life cycle (aeciospore, urediniospore, and mycelium growth inside cankered pine stem) except that CrMV2 was not detected in fungal isolate OR-res1. In contrast to RNA from cankered pine stems infected by *C*. *ribicola*, RNA samples from healthy pine stems were consistently free of any CrMV. Prevalence of the viruses was further confirmed by regular RT-PCR. Viral cDNA fragments were amplified with expected sizes of 398-bp to 547-bp for each of the five viral genomes in 14 fungal isolates, but no viral cDNA amplicon was detected in isolate OR-res1.

**Fig 4:**
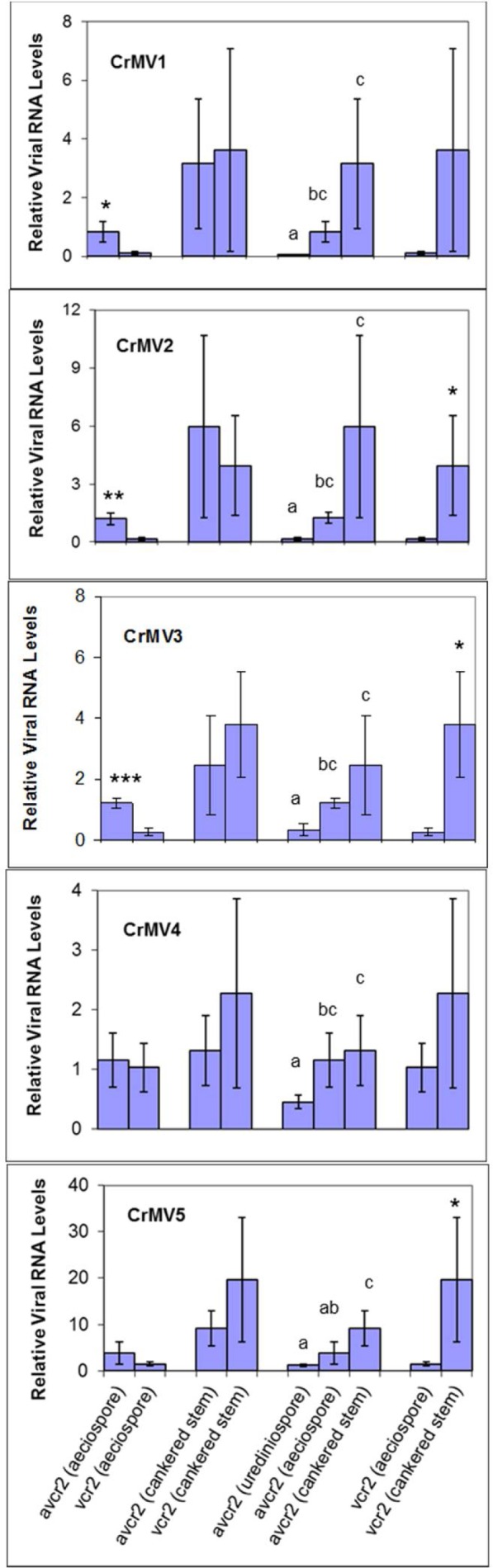
Genomic RNA abundance of *Cronartium ribicola* mitoviruses (CrMV1 to CrMV5) as measured by qRT-PCR analysis. *C*. *ribicola tubulin* transcript was used as the calibrator for normalization of input RNA levels across the 15 fungal isolates that are listed in Table 1. For each mitovirus, RNA levels in different fungal isolates were calculated according to the 2^−ΔΔCt^ algorithm using *C*. *ribicola* isolate BC-a6 as the reference. Means for relative levels of viral RNA accumulation in each type of samples are shown. Bars with different letters were significantly different using t-test (*P* <0.05). One, two, and three stars (*) indicate *P* <0.05, *P* <0.01, and *P* <0.001, respectively. Four comparisons were made as: (1) *avcr2* isolates (BC-a6, BC-a20, and BC-a28) *vs*. *vcr2* isolates (OR-a1, OR-a2, and OR-a3) at the aeciospore stage; (2) *avcr2* isolates (OR-sus1, OR-sus2, and OR-sus3) *vs*. *vcr2* isolates (OR-res1, OR-res2, and OR-res3) at the mycelium growth stage inside the cankered stem; (3) *avcr2* isolates at the stage of aeciospores (BC-a6, BC-a20, and BC-a28) *vs*. urediniospore (BC-u2a, BC-u3, and BC-u48), and *vs*. the stage of mycelium growth inside cankered stem (OR-sus1, OR-sus2, and OR-sus3), and different letters indicate significant differences between groups (p<0.05); (4) *vcr2* isolates at the stage of aeciospore (OR-a1, OR-a2, and OR-a3) *vs*. the stage of mycelium growth inside cankered stem (OR-res1, OR-res2, and OR-res3).

Average viral abundance was compared between *C*. *ribicola* avirulent isolates (*avcr2*) and virulent isolates (*vcr2*) at either aeciospore or mycelium growth stage, as well as between life cycle stages of *avcr2* or *vcr2* isolates. Analysis of qRT-PCR data demonstrated no significant difference of viral RNA levels between *avcr2* and *vcr2* isolates for all five CrMVs at mycelium growth stage in cankered pine stems. However, CrMV1, CrMV2 and CrMV3 accumulated at significantly higher levels (fold change 4.7~8.0) in *avcr2* isolates than in *vcr2* isolates at the aeciospore stage (*P* < 0.05) (Fig 4A), suggesting that fungal isolate genotypes may affect spore-mediated virus transmission for some mitoviruses.

Comparisons among three of the life cycle stages of *avcr2* isolates showed that the fungal cells at the urediniospore stage (on *Ribes* leaves) contained CrMVs at levels significantly lower than those at the stages occurring inside pine stem tissues (aeciospore fold change 2.6~17.4, mycelium growth fold change 2.9~67.4) for all five CrMVs (*P* < 0.05), except for CrMV5 compared between urediniospores and aeciospores (Fig 4B). With the exception of CrMV4, CrMVs were more abundant in *avcr2* mycelium (fold change 2.1 ~5.0) than in *avcr2* aeciospores, but the fold changes were not significant due to high variation of viral RNA levels among isolates at the same life cycle stage (Fig 4B). Fungal *vcr2* isolates showed a similar viral accumulation pattern as *avcr2* isolates: CrMVs were more abundant (fold change 2.2~35.1) at the mycelium growth stage than those at the aeciospore stage, with statistically significant differences in RNA levels for CrMV2, CrMV3, and CrMV5 (*P* < 0.05) (Fig 4C). All of these observations suggest that plant host may be one of the key factors affecting mitoviral accumulation in the fungal component of the WPBR pathosystem.

### Up-regulation of CrMV transcripts during mycelium growth inside cankered pine stems

**Fig 5:**
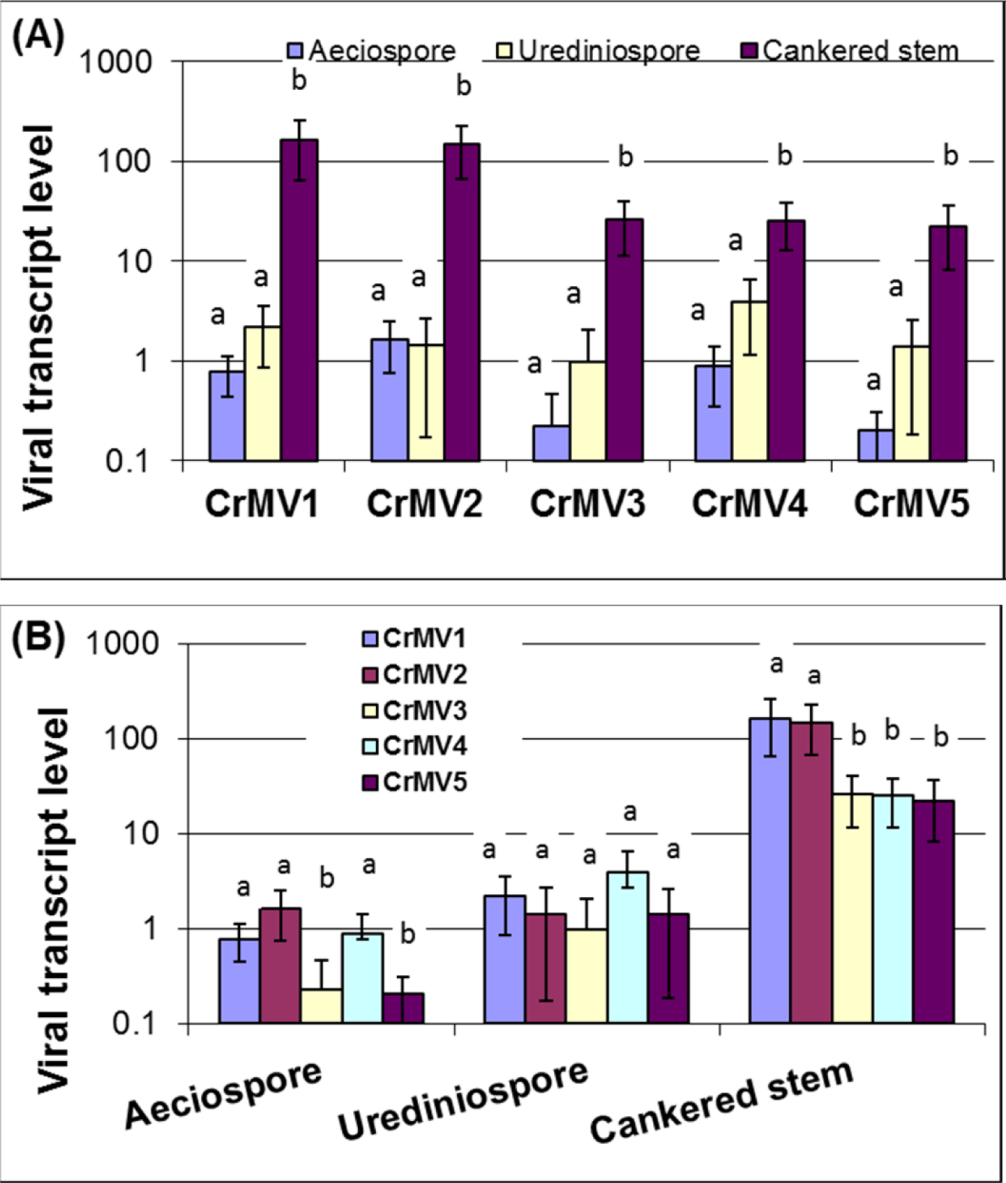
Relative transcript levels of *Cronartium ribicola* mitoviruses (CrMV1 to CrMV5) as measured by FPKM in RNA-Seq analysis at three fungal life cycle stages in *avcr2* isolates. Bars with different letters were significantly different using t-test (*P* <0.05). (A) Comparison of relative transcript levels of each mitovirus at three stages of the fungal life cycle; (B) Comparison of relative transcript levels of the five mitoviruses at each of the three selected fungal life cycle stages

Complete coverage of all mRNA expressed in sampled tissues is conceivable using transcriptome profiling by RNA-Seq. Given that a viral ssRNA(+) genome is in the form of mRNA, mapping of deep mRNA sequencing reads from samples collected at different times and in different places would characterize spatiotemporal expression and replication of ssRNA(+) genomes or viral transcripts. Our counts of mapped RNA-Seq reads revealed that average coverage varied greatly among the five CrMV genomes across the nine *avcr2* isolates (S3 Table). Coverage ranged from 0 (CrMV3 in isolate BC-6) to 405 times (CrMV1 in isolate OR-sus2). FPKM was further used to evaluate relative levels of viral transcripts or ssRNA(+) genomes (Fig 5). In general, both genome coverage and FPKM analyses revealed a pattern similar to that detected by qRT-PCR analysis. Fungal isolates (OR-sus1, OR-sus2, and OR-sus3) from the mycelium growth stage showed much higher viral coverage or FPKM values compared to fungal isolates from the aeciospore or urediniospore stage. Relative viral transcript levels of all five CrMVs were significantly increased in mycelia (fold change 6.6 ~ 209.9) compared to that in either aeciospores or urediniospores (*P* <0.05), but no significant difference was detected between the two fungal spore stages (Fig 5A). The relative transcript / ssRNA(+) levels of CrMVs measured by FPKM were correlated to the total RNA (dsRNA and ssRNA) mitovirus levels measured by qRT-PCR (R = 0.6003) with statistical significance (*P* = 1.3E-05) (S1A Fig). Fungal isolates at the mycelium growth stage from infected white pine stems showed better correlation between FPKM and qRT-PCR results. Fungal isolates at the spore stages, especially aeciospores, showed relatively high total RNA levels as compared to ssRNA level (S1B Fig), which suggests that a large amount of viral RNAs may be present in a form of dsRNA in fungal spores.

As FPKM values were normalized by the whole fungal reference transcriptome, it allowed comparison of different viruses in the same sample, (Fig 5B). At the aeciospore stage (isolates BC-a6, -a20, and -a28), relative transcript levels of CrMV3 and CrMV5 were significantly lower (fold change 3.4 ~ 8.1) than the other three viruses (*P* <0.05). At the mycelium growth stage (isolates OR-sus1, OR-sus2, and OR-sus3), CrMV1 and CrMV2 showed relative transcript levels significantly higher (fold change 5.7 ~7.2) than the other three CrMVs (*P* <0.05). However, there was no significant difference of transcript levels among the five CrMVs at the urediniospore stage (isolates BC-u2a, -u3, and–u48) (Fig 5B).

### SNP detection and genetic diversity

**Fig 6:**
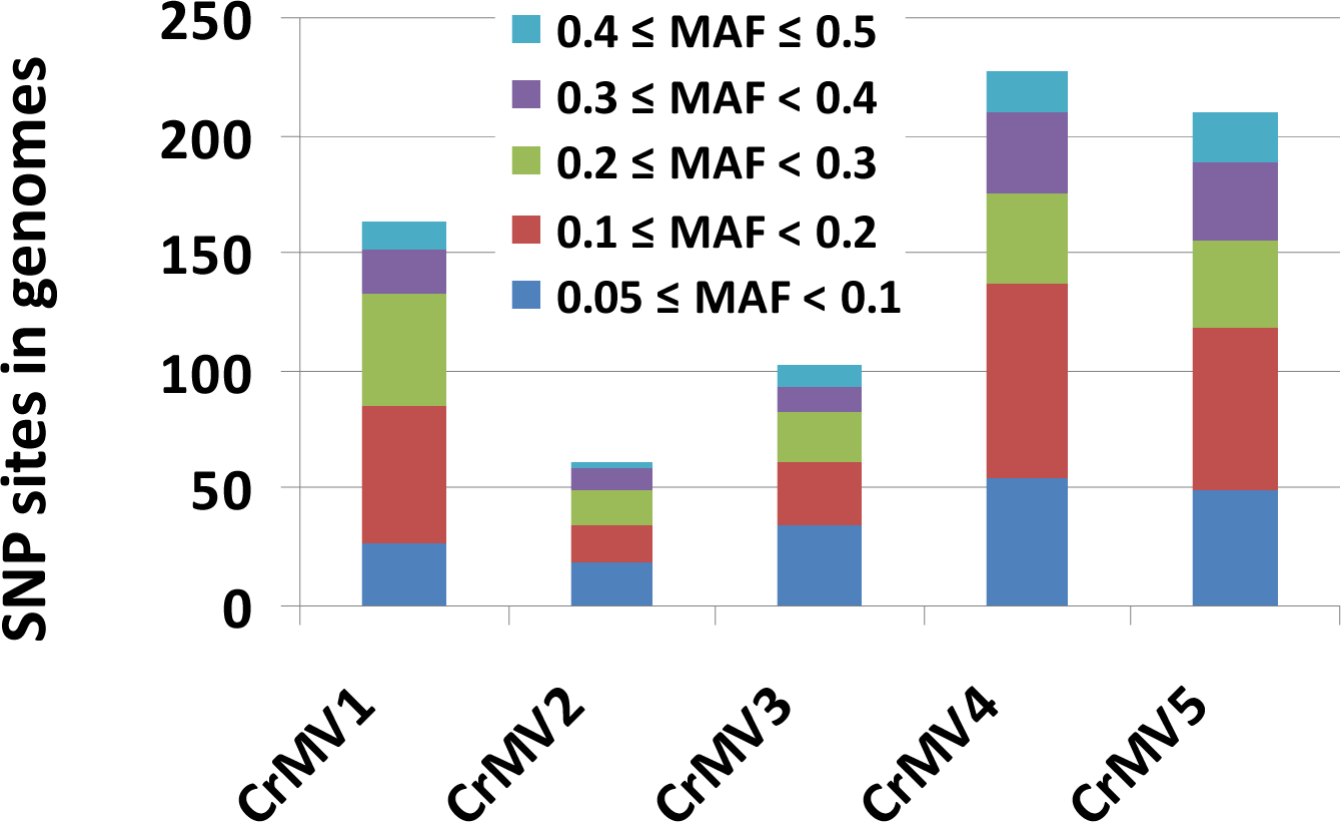
Distribution of minor allele frequency (MAF) of single nucleotide polymorphism (SNP) sites in the genomes of *Cronartium ribicola* mitoviruses (CrMV1 to CrMV5) as detected by RNA-Seq analysis.

Mycoviral populations are expected to be heterogeneous in time and space. We detected SNPs and genetic diversity of CrMVs using RNA-Seq read data. Minor allele frequency (MAF) was calculated based on SNP sites detected in the viral genomes. As only variants with MAF ≥0.05 were regarded to be true, those with MAF ≥0.05 and coverage ≥10 were further analyzed. SNP frequencies (variant sites per 100-bp) ranged from 1.82% (CrMV2) to 7.02% (CrMV4) (Table 3). Investigation of MAF distribution found that the five CrMVs showed a similar pattern: 74%~81% of total SNPs displayed a MAF ranging from 5% to 30% (Fig 6), from which we inferred that fungal isolates were co-infected with multiple strains of the same mitovirus species with minor viral strains at intermediate frequencies in the populations.

**Table 3:** Genomic variations of *Cronartium ribicola* mitoviruses (CrMVs) in BC and OR regions.

Virus ID	SNP types	BC-Specific	OR-Specific	BC-OR Shared	Total	Frequency of SNP (%)	AA change rate (%)
**CrMV1**	Nonsynonymous	2	3	3	8		
** **	Synonymous	18	52	41	111		
** **	5'- or 3'-UTR	0	1	0	1		
** **	Total	20	56	44	120	4.42%	1.00%
**CrMV2**	Nonsynonymous	4	1	2	7		
** **	Synonymous	9	13	13	34		
** **	5'- or 3'-UTR	1	1	1	3		
** **	Total	14	15	16	45	1.82%	1.02%
**CrMV3**	Nonsynonymous	2	5	1	8		
** **	Synonymous	8	70	5	83		
** **	5'- or 3'-UTR	0	6	0	6		
	Total	10	81	6	97	3.85%	1.12%
**CrMV4**	Nonsynonymous	13	7	7	27		
** **	Synonymous	37	58	46	141		
** **	5'- or 3'-UTR	3	2	1	6		
** **	Total	53	67	54	174	7.02%	3.74%
**CrMV5**	Nonsynonymous	3	15	10	28		
** **	Synonymous	21	87	30	138		
** **	5'- or 3'-UTR	1	3	0	4		
** **	Total	25	105	40	170	6.46%	3.63%

Of 605 total annotated SNPs in the five CrMV genomes, most were located in RdRp-coding regions, including 78 non-synonymous SNPs (nsSNPs) and 507 synonymous SNPs, while 20 SNPs were located in 5′ or 3′ UTR regions (Table 3). As amino acid change may affect RdRp activity directly, we examined the nsSNPs in each viral genome. Amino acid change rates ranged from 1.00% (CrMV1) to 3.74% (CrMV4) (Table 3), suggesting that genetic divergence is limited in sequenced populations for individual CrMV species. Geographically, nsSNP sites in CrMV1 were similar in the BC and OR region (5 vs. 6). CrMV2 and CrMV4 had more nsSNP sites in BC than in OR (6 vs. 3, and 20 vs. 14) and CrMV3 and CrMV5 had fewer nsSNP sites (3 vs. 6, and 13 vs. 25) in BC than in OR, indicating genetic divergence of CrMVs between BC and OR regions.

## Discussion

### RNA-Seq-based discovery of novel mycoviruses in the WPBR pathosystem

Recent advances in next generation sequencing (NGS) technologies and bioinformatics provide a potential for the discovery of new species or isolates of known virus families or novel viruses with little sequence similarity to any reported viruses [37]. The current lack of an effective method for *in vitro* culture of *C*. *ribicola* makes it difficult to both identify mycoviruses in the WPBR pathosystem and explore their potential effects in the plant-fungus-virus interaction. A growing number of case studies have demonstrated NGS to be a powerful technique for cataloguing a virome. This method has the potential to cover all viruses and viroids in an environmental sample, irrespective of whether their hosts are culturable or unculturable [38]. The availability of a large number (10^7^ ~10^9^) of shotgun sequences makes an NGS approach sensitive enough to detect viruses at low titre as well as viral genomic variation at low MAFs [39]. Current NGS data suggest that the known viruses may only account for < 1% of the viral world [38]. Therefore, the use of bioinformatics for *de novo* assembly and data mining has an important and increasing application in the discovery of novel viruses [11]. Omics resources and research tools have advanced various areas of virology studies, including viral genome sequencing, ecology, epidemiology, replication, transcription, genome variability, and viral evolution [40, 41].

We used a deep mRNA sequencing approach to generate large amounts of RNA-Seq raw reads from cDNA libraries, representing the transcriptomes of multiple organisms (plant, fungus, and virus) involved in the WPBR pathosystem—a pathosystem which currently has limited genomic and transcriptomic data [29, 31]. In the WPBR metatranscriptomic data, mitoviruses were detected in eight of nine *C*. *ribicola* isolates by BLASTx searching against the known viral database (Table 1). This metatranscriptomic survey also identified additional contigs with homology hits to RdRp of other virus families (data not shown), suggesting presence of mycoviral sequences other than those of mitoviruses in the WPBR pathosystem. Complete genomic sequences of five *C*. *ribicola mitoviruses* (CrMV1 to CrMV5) were then determined in a BC *avcr2* isolate by RACE-based cDNA cloning and Sanger re-sequencing (Table 1).

Species in the genus *Mitovirus* have a few common structural features: ss-RNA(+) genome with relatively high A/U content (∼62%–73%), a single large ORF encoding for an RdRp with usage of the mitochondrial genetic codes, and stable secondary structures potentially formed at the 5′- and 3′-termini of the genome [2]. Based on these structural features, we assigned CrMVs to the genus Mitovirus, which is further supported by the alignment analysis of RdRp sequences (Fig 1) and phylogenetic analysis (Fig 2). According to the International Committee on Taxonomy of Viruses (ICTV) regarding sequence-related criteria for delineation of species in the genus Mitovirus, viruses are considered as different species if their RdRp sequence identities are < 40%, while different strains of the same species share RdRp sequence identities > 90% [2]. Due to low RdRp identities to reported mitovirues (≤ 28.37%) and among the five CrMVs (≤ 35.13%), we thus propose that *C*. *ribicola* mitoviruses (CrMV1 to CrMV5) are five novel species of the genus *Mitovirus* in the family *Narnaviridae*. To the best of our knowledge, this is the first report of naturally occurring mitoviruses infecting a rust fungus in the Class *Pucciniomycetes*. Characterization of the mitovirus-fungus interaction in the WPBR pathosystem may advance our understanding of the ecology and evolution of mitoviruses in the family *Narnaviridae*.

### Transcriptome profiling for understanding host-virus interaction

In recent years a small number of plant RNA viruses and viroids have been identified by deep sequencing various types of RNAs, including total RNA, dsRNA, mRNA, and small/short interfering RNA (siRNA) from infected tissues [41]. Transcriptome profiling has the potential to discover viral mRNAs transcribed from viral genomes, or ssRNA(+) genomes directly, from an environmental sample. Direct deep mRNA sequencing has been used to detect several novel virus species in a few diseased or symptomless plants [39, 42, 43]. Our RNA-Seq study revealed multiple viral transcripts or ssRNA(+) genomes in the transcriptomes of eight *C*. *ribicola* isolates. A previous study also reported detection of novel RNA mycoviruses, including a mitovirus (HfMV1), in the pathogenic fungus *Hymenoscyphus fraxineus* by deep mRNA sequencing [11].

Compared to siRNA or dsRNA deep sequencing, deep mRNA sequencing may provide more information for understanding virus-host interactions, as both host and pathogen transcriptomes are profiled from the same sample. The number of NGS raw reads is commonly used to infer the relative abundance of different viral species in a community as the shotgun reads mapped to the templates are statistically in proportion to population frequency [44]. In order to obtain an initial insight of mitoviral activity, viral transcripts or ssRNA genomes were analyzed in *C*. *ribicola* isolates using Illumina RNA-Seq read data. Our RNA-Seq-based survey of host samples at multiple fungus life cycle stages led to the discovery of these novel mitoviruses in the WPBR pathosystem, due to the conspicuously high variation of virus titers among *C*. *ribicola* isolates. Based on mapped read counts, RNA-Seq assays revealed differential expression of viral transcripts in fungal spore and fungus-infected plant tissues (Fig 5). The differential transcriptional activity was generally consistent with the abundance of total viral RNA (including dsRNA and ssRNA) as detected by qRT-PCR (Fig 4). All five CrMVs were detected with low mRNA or ssRNA(+) levels at the aeciospore stage, which may limit virus transmission from white pines to Ribes. Concordantly, we observed low but even levels of transcript/ssRNA(+) across five CrMVs in fungal urediniospores sampled from infected *Ribes* leaves. These results suggest that CrMV transcription may not be differentially affected by *Ribes*-rust interactions.

In contrast, viral transcriptional activity was observed at high levels at the mycelium growth stage in cankered pine stem tissues. CrMV1 and CrMV2 ssRNA(+) sequences were revealed with FPKM >100; among the most abundant transcripts in the *C*. *ribicola* transcriptome. Fungal metabolic pathways were widely reprogrammed for infection in plant tissues during *C*. *ribicola* life cycle; and the transcription process was significantly increased at mycelium growth stage [29]. This up-regulated transcription machinery may be necessary for active viral replication in all tested isolates because no significant difference of viral RNA levels were detected between *avcr2* and *vcr2* isolates for all five CrMVs at the mycelium growth stage. Enhanced viral productivity in *C*. *ribicola*-white pine interactions has important implications for the study of canker development in WPBR disease dynamics.

Differential accumulation of CrMVs in *C*. *ribicola* isolates and their prevalence throughout the hosts’ life cycle indicate an extensive diversity of intrahost viral community structures. However, we do not know the molecular interactions underlying CrMV dynamics in the WPBR pathosystem. Currently it is very difficult to separate and culture *C*. *ribicola* isolates. Biological traits such as hypovirulence and hypervirulence have not been characterized in *C*. *ribicola* populations. It awaits a future study to determine whether the presence of a CrMV or the CrMV burden can have an important role in pine stem canker progression. A survey of mitoviruses across the *C*. *ribicola* landscape, including virulent (*vcr1* and *vcr2*) and avrirulent (*avcr1*-*avcr4*) isolates of the fungus, would provide novel insight into the potential effects of mitoviruses on the biology/virulence of *C*. *ribicola* (e.g. mitochondrial abnormalities in mitovirus-infected spores, virulence on black currants and on other five-needle pines of virus-free or mitovirus-infected spores). Mitovirus-like sequences may transfer between fungi to host plants [45], thus serving as a plant defense mechanism against widespread and devastating fungal pathogens by reducing fungal virulence [46]. Further spatiotemporal characterization of viral transcripts across the *C*. *ribicola* landscape would help unravel details about viral replication, transmission, and the molecular interaction of plant-fungus-mycovirus-environmental factors in the WPBR pathosystem.

### Genetic diversity of *C*. *ribicola* mitoviruses

Information about mycoviral diversity and evolution would facilitate management of plant pathogenic fungi. We investigated genomic variations of CrMVs by SNP detection and MAF analysis, with a focus on nsSNPs due to their potential to directly influence the RdRp activity. RdRps are responsible for genome replication, mRNA synthesis, RNA recombination, RNA silencing, and other biological processes in RNA viruses. RdRps are also crucial to viral genome variability and evolution, because they have high error rates (average ~10^−4^) in viral RdRp copying. This wide variability results in high genetic diversity in RNA virus populations, allowing rapid virus evolution under selective pressures imposed by the host immune response and other anti-viral factors [47]. We mapped RNA-Seq reads of fungal isolates back to each CrMV genome. High coverage of viral genomes allowed detection of high quality SNPs in BC and OR regions. nsSNPs resulted in frequencies of amino acid change from 1.00% to 3.74% among RdRps of CrMVs (Table 3), and these snSNPs provide valuable candidate sites for further genome wide association studies when hypovirulent isolates are available.

The secondary structures at the 5’- and 3′-UTRs are important for initiation of mitoviral genome replication [12, 15]. SNPs in 5′- or 3′-UTRs are also of interest since some of them might lead to changes in the secondary structures, thereby affecting viral replication and translation. Based on variation sites of viral genome sequences, biomarkers and genomic tools may be developed with potential applications in investigating the population structure of mycoviral communities and monitoring dynamic changes in the spread of both mycoviruses and their host pathogenic fungi [48].

We found that most SNPs (74%~81% of the total) in CrMV genomes have a MAF below 30%, suggesting the occurrence of multiple minor viral strains in *C*. *ribicola* populations. These polymorphic genome sequences could be used to investigate population diversity or population differentiation for mitoviruses and rust fungal isolates in the WPBR pathosystem. *C*. *ribicola* virulence factors are known to have a genetic association with cytoplasmic inheritance [30]. A number of mitoviruses have been reported in association with hypovirulence [24], which provides great potential as biocontrol agents for plant pathogenic fungi. Three-way interactions within the virus-fungus-plant trio may benefit survival and growth of plants [49]. Co-infection of multiple mitoviral species and relatively high genetic diversity of each CrMV species also present an opportunity to select viral variants that cause reduced virulence in the host fungus and thus be suitable for development of a novel management tool for control of *C*. *ribicola*. The relationship between the genetic variability of CrMVs and the pathogenicity of *C*. *ribicola* needs to be investigated. A further ecological study of mycoviruses, including their phenotypic and genetic diversity, transmission and evolution, could help improve biocontrol strategies for fungal diseases. Elucidation of molecular mechanisms underlying the association of mycoviruses with virulence and pathogenicity in the plant pathogens will provide a means for engineering mycoviruses for enhanced biocontrol potential [50].

In conclusion, five novel mitoviruses have been discovered with relatively high genetic diversity in *C*. *ribicola* populations. Analysis of full genome sequences demonstrated that CrMVs were present as ssRNA(+) and dsRNA and phylogenetically related to other species previously reported in the genus *Mitovirus*. Viral transcript analysis revealed that CrMVs were highly expressed at the fungal mycelium growth stage inside the pine stem tissues where the rust fungus was propagating actively. Transcriptomes of *C*. *ribicola* and five-needle pines are becoming available, which increases the potential to better understand mycovirus-fungus-plant interactions. This first report of mycoviruses infecting *C*. *ribicola* sets the stage for further study of the diversity and biology of mycoviruses in pathogenic basidiomycete fungi.

## Supporting Information

S1 Fig Comparison of relative RNA levels as detected by FPKM based on RNA-Seq analysis and quantitative reverse-transcriptase-polymerase chain reaction (qRT-PCR). RNA levels of five mitoviruses in nine fungal *avcr2* isolates were included. (A) Scatter-plot to show all data of relative transcript levels measured by FPKM (y axis) and relative total RNA levels detected by qRT-PCR (x axis). Isolates at the mycelium growth stage (InS) from infected white pine stems are circled. (B) Bar-plot to show relative RNA levels of five mitoviruses (CrMV1 to CrMV5) in aeciospores (Aec), urediniospore (Ure), and mycelium at growth stage inside infected white pine stems (InS).
(TIFF)

S1 Table Summary of information for fungi known to host mycoviruses. (XLSX)

S2 Table Primers used for investigation of *Cronartium ribicola* mitoviruses. (XLSX)

S3 Table RNA-Seq read count and relative coverage of *Cronartium ribicola* mitoviruses. (XLSX)
