# Supplementary figures and images for: Characterization of Five Novel Mitoviruses in the White Pine Blister Rust Fungus Cronartium ribicola
Source: PLoS One. 2016 May 19;11(5):e0154267. doi: 10.1371/journal.pone.0154267 (PMC4873031; doi:10.1371/journal.pone.0154267)

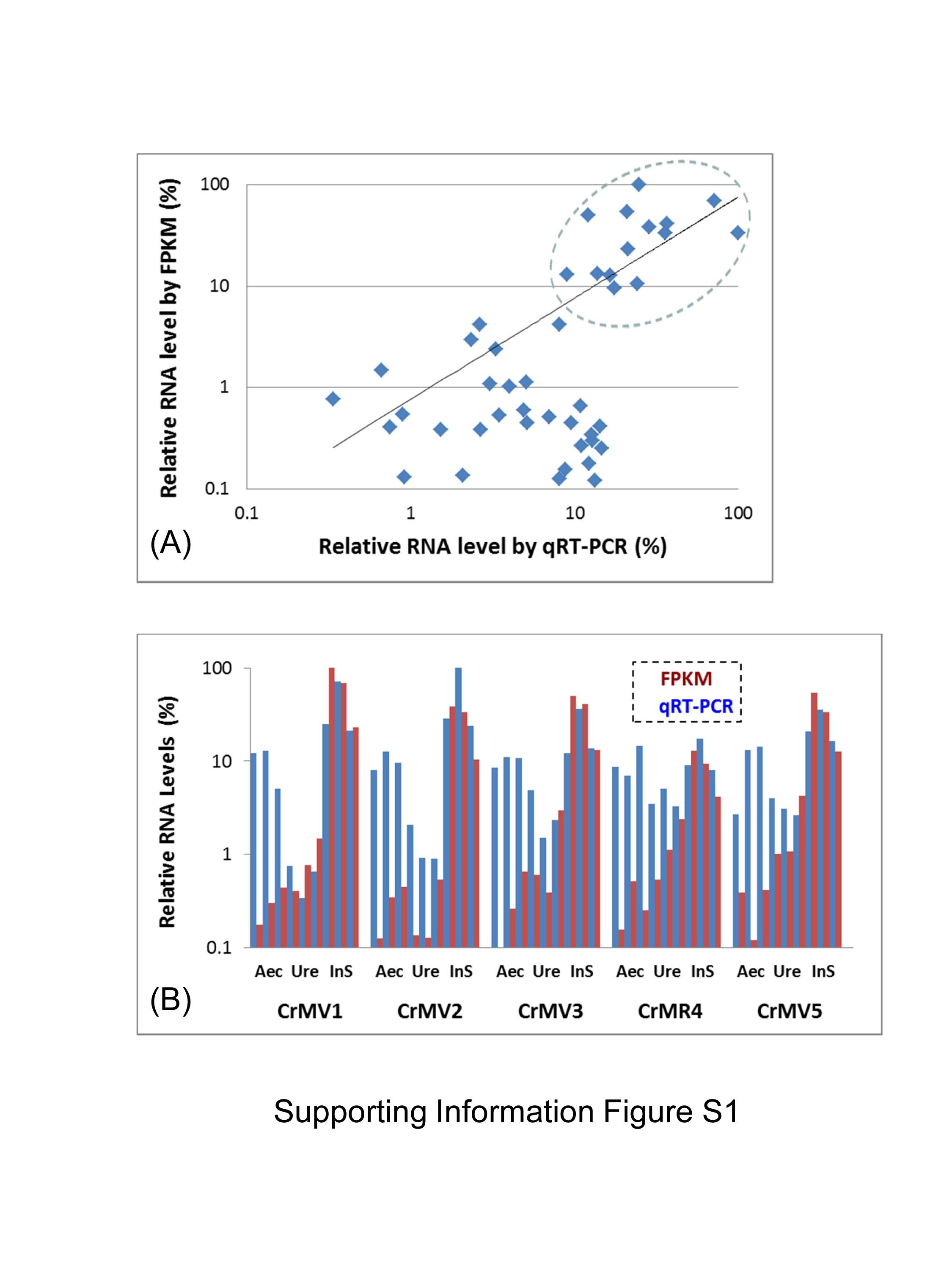

Supplement: S1 Fig — RNA levels of five mitoviruses in nine fungal avcr2 isolates were included. (A) Scatter-plot to show all data of relative transcript levels measured by FPKM (y axis) and relative total RNA levels detected by qRT-PCR (x axis). Isolates at the mycelium growth stage (InS) from infected white pine stems are circled. (B) Bar-plot to show relative RNA levels of five mitoviruses (CrMV1 to CrMV5) in aeciospores (Aec), urediniospore (Ure), and mycelium at growth stage inside infected white pine stems (InS). (TIFF) [file pone.0154267.s001.tiff]
